# Imaging ovarian cancer – from baseline characteristics to high-risk image factors

**DOI:** 10.1186/s13048-023-01154-w

**Published:** 2023-04-17

**Authors:** Hanna Sartor, Maria Bjurberg, Mihaela Asp, Anna Kahn, Jenny Brändstedt, Päivi Kannisto, Karin Jirström

**Affiliations:** 1 Department of Translational Medicine, Diagnostic Radiology, Lund University, Skåne University Hospital, Lund, Sweden; 2 Department of Clinical Sciences Lund, Oncology and Therapeutic Pathology, Lund University, Lund, Sweden; 3 Department of Clinical Sciences Lund, Obstetrics and Gynecology, Lund University, Lund, Sweden

**Keywords:** Ovarian cancer, Peritoneal Carcinomatosis, Lymph nodes, Ovarian cancer survival

## Abstract

**Background:**

Imaging ovarian cancer (OC) includes evaluating peritoneal carcinomatosis (PC) and enlarged cardio phrenic lymph nodes (CPLN) by computed tomography (CT), and thorough evaluation is tedious work. A “CT short score” with high-risk CT parameters might be a more pragmatic approach, but it is not known if such a short score associates with aggressive OC subtypes and impaired OC survival. Further, it is not known if certain established OC risk factors are linked to high-risk CT-findings which would be important in image evaluation. Herein, we investigate a CT short score and its relation to baseline characteristics, OC subtypes, and survival.

**Methods:**

The Malmö Diet and Cancer Study is a prospective cohort that included 17,035 women (1991–1996). Baseline characteristics and tumor information on 159 OC and information on OC specific survival (last follow-up, 2017-12-31) was registered. A CT short score (CPLN and PC-index (PCI) in seven regions) was registered and associations with clinical stage [stage I vs. advanced stage (II-IV), histological type/grade (high grade serous and endometrioid vs. other subtypes], and OC-specific survival were analyzed with logistic and Cox regression, respectively. Parity and menopausal status were analyzed in relation to short score and PCI.

**Results:**

There was an association between higher short score and advanced clinical stage (adjusted OR 2.76 (1.42—5.38)), adjusted for age at diagnosis and histological type/grade. Higher short score was associated with impaired OC specific survival (adjusted HR 1.17 (1.01—1.35)), adjusted for age at diagnosis, histological type/grade, and clinical stage. There were no significant associations between parity, menopausal status, and short score/PCI.

**Conclusions:**

CT short score was significantly associated with advanced clinical stages and impaired OC survival. A pragmatic approach (based on CT) to evaluate high risk image findings in OC could help reduce radiologists’ workload and at the same time provide structured reports to surgeons and oncologists involved in OC care.

## Introduction

Computer tomography (CT) is an established tool in ovarian cancer (OC) diagnosis, clinical decision making regarding surgery and treatment, and follow-up of cancer survivors [1].

The CT provides highly accurate information on ovarian tumor load and abdominal spread (i.e. peritoneal carcinomatosis) [2, 3]. However, the radiological description and presentation is subjective and very much depending on radiologists and local traditions. The established peritoneal carcinomatosis index [4] (PCI) is a structured score which evaluates and quantifies the carcinomatosis in CT (CT-PCI) and/or at surgery (surgical-PCI (S-PCI)).

A recent paper from our research group showed that high CT-PCI was associated with higher clinical stage and impaired OC survival [5], which is in line with previous studies also highlighting the value of CT-PCI in evaluating surgical outcome [6] and patient prognosis [7]. However, from a radiological perspective, a thorough PCI-scoring is a tedious procedure not prioritized in daily clinical work. A previous paper on S-PCI concluded that selected PCI regions (i.e., small intestine and hepatoduodenal ligament) were more predictive of complete resection and survival than the entire PCI-score [8]. With support in previous research, we therefore hypothesize that an abbreviated CT-based image short score might offer the same clinical insights as the full CT-PCI, and at the same time be clinically feasible, and this has to the best of our knowledge not been previously studied. In addition, such a score could include additional high-risk image parameters (e.g., enlarged lymph nodes) and not be limited to carcinomatosis.

At the very beginning of the diagnostic chain, primary prevention is a goal of future OC care. As of today, it is not known whether any of the established OC risk factors in healthy women may lead to high-risk image factors in the later established OC diagnosis. A previous study by Poole et al. (*n* = 4342 OC cases) investigated differences in the associations with OC risk factors by tumor aggressiveness (rapidly fatal (dead within three years) vs all other tumors) and concluded that rapidly fatal cases were older and tended to have shorter duration of oral contraceptive use [9]. A similar approach but with imaging as endpoint (high risk image being defined as increasing CT-PCI or CT-based short score) has to the best of our knowledge not been studied. Understanding and highlighting such risk factors may add knowledge to how patient factors are potentially linked to the image. Further, deepened knowledge could implicate that the radiologist should pay extra attention to the presence or absence of a particular risk factor at time of the CT reading.

The goal of this study was to analyze a CT-based image short score, with the hypothesis that this would provide similarly strong, or stronger, associations with OC prognosis as the PCI-score. In addition, we wanted to explore if any of the known OC risk factors was linked to image factors included in the score.

## Methods

### The Malmö diet and Cancer study (MDCS)

The MDCS [10–12] is a population-based, prospective cohort study, which included 17,035 women during 1991–1996. Patient related data was registered by trained nurses at inclusion (in this study specifically parity, nulliparity, oral contraceptives, height, weight, body mass index (BMI), menstrual status, hormone replacement therapy). Incident OC cases were identified prospectively through linkage with the National Cancer Registry. OC specific pathological variables (histological subtype and grade, clinical stage) and radiological variables (CT-PCI and CPLN) from time of diagnosis were collected and added to the database retrospectively for research purposes. Cause of death (OC as an underlying or subordinate cause of death) and vital status (alive or dead from another cause was classified as alive) was registered with the last follow-upon 31 December 2017 (mean follow up time, 16.7 years). The MDCS (LU 51–90) and the present study (Dnr 530/2008) were approved by the regional ethics committee in Lund, Sweden. All women gave written informed consent at study inclusion.

### CT

Only patients with digital CT scans were eligible for analyses. Hence, patients with analogue CT images are classified as “missing” in image variables (Table 1). The median time between diagnostic CT and registered OC diagnosis was 6 days (range 152 days). All patients underwent CT in the supine position and the majority with intravenous and oral contrast and with images reformatted in the coronal and sagittal planes. The images were gathered over a long period of time. Hence, there is a variety of radiology systems; this has, however, been shown not to affect peritoneal carcinomatosis detection [2].

**Table 1:** Patient and tumor characteristics

	n	159
Age at diagnosis	median (range)	68 (40)
	Missing	0
Height, cm	median (range)	164 (29)
	Missing	0
Weight, kg	median (range)	65 (57)
	Missing	0
BMI	median (range)	24 (20)
	Missing	0
Menstrual status	Pre	39 (24.5)
	Peri	13 (8.5)
	Post	107 (67.3)
	Missing	0
Parity	median (range)	2 (4)
	Missing	3
Nullipara	No	125 (80.1)
	Yes	31 (19.9)
	Missing	3
HRT	No	119 (74.8)
	Yes	40 (25.2)
	Missing	0
Oral contraceptives	No	85 (53.5)
	Yes	74 (46.5)
	Missing	0
Ovarian cancer death	No	58 (36.5)
	Yes	101 (63.5)
	Missing	0
Stage	Stage I	26 (16.8)
	Advanced Stage	129 (83.2)
	Missing	4
Histological type/grade	Low grade serous/other	46 (29.9)
	High grade serous and all endometroid	108 (70.1)
	Missing	5
PCI	median (range)	10.5 (39)
	Missing	65
Short score	median (range)	2 (7)
	Missing	65
CPLN	< 5 mm	46 (48.9)
	≥ 5 mm	48 (51.1)
	Missing	65

### CT-PCI and CPLN

CT-PCI was retrospectively scored using the Sugarbaker classification [3, 4] by one specialist in radiology with subspeciality training in gastroradiology (CB). The CT-PCI was defined as the sum of numerical lesion scores in 13 abdominopelvic regions. The lesion score relates to the largest visible deposit. CPLN was considered pathological if measuring ≥ 5 mm in the short axis in the axial plane [13] and was defined as negative or positive (i.e., enlarged). The evaluation of CPLN was made using the same CT as for CT-PCI and was retrospectively analyzed by one radiologist with subspecialty training in gastroradiology (AK).

### CT short score

**Fig. 1:**
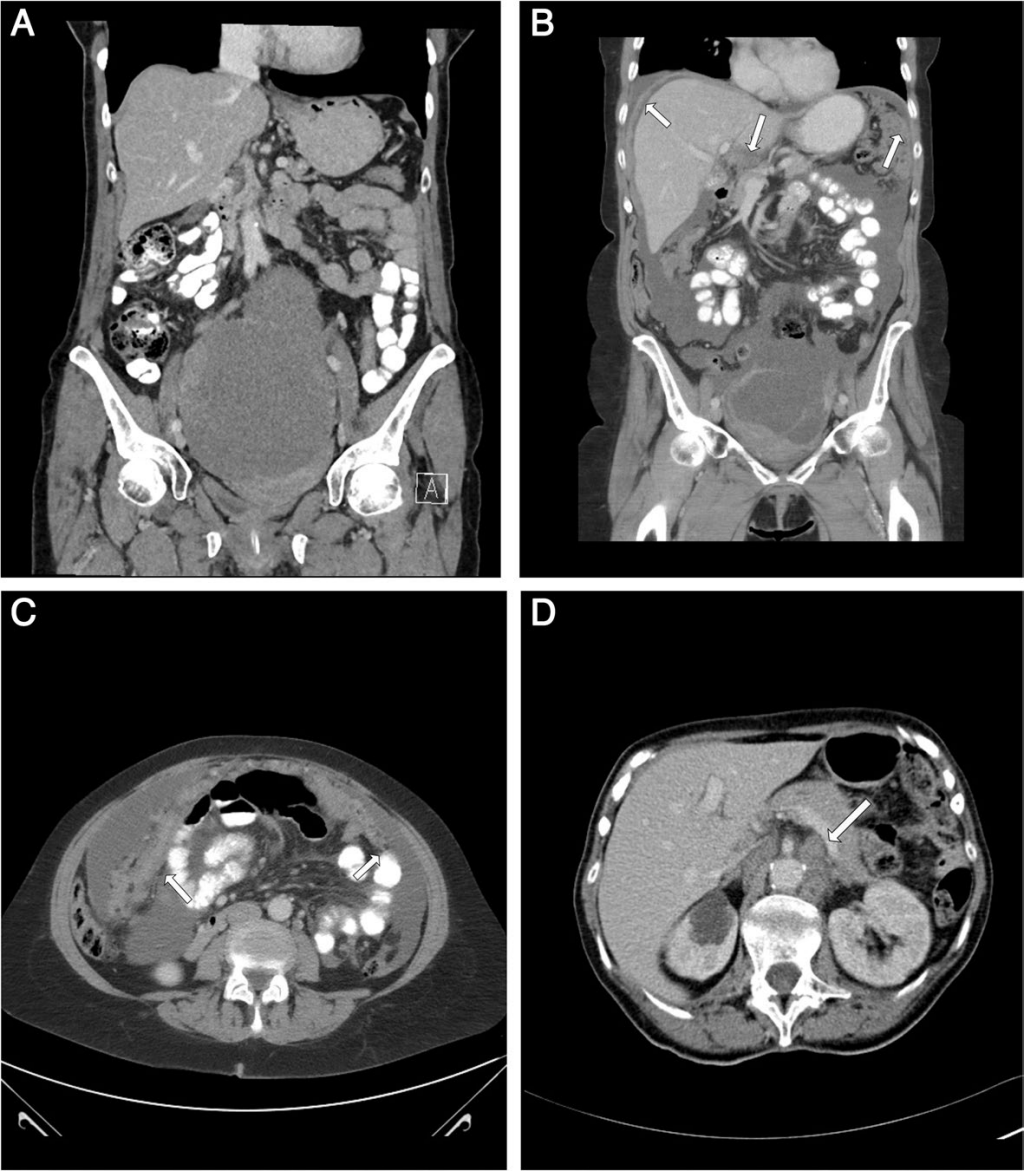
**A** A coronal abdominal CT showing a left sided ovarian tumor mass, however no signs of additional spread in the abdomen (0 points). **B** (coronal) and **C** (axial), same patient (6 points): An abdominal CT showing involvement of right and left diaphragm (2 points), liver hilum (1point), small intestines (1 point, image C), coeliac trunc (1point) (not shown in image), and cardiophrenic lymph nodes (not shown in image) (1 point). **D** An axial abdominal CT showing involvement of coeliac trunc and lymph nodes above the renal artery level, a short score of 2 points

The novel CT short score can be seen as an abbreviated variant of the established CT-PCI (with previously shown high intra- and inter-observer reliability) [3]. The definition of the short score was made by two specialists in gynecological tumor surgery (MA and PK) based on previous research [8] and their own clinical experience of surgically difficult areas in OC surgery. The short score evaluates carcinomatosis and pathological lymph nodes in seven regions in the abdominal CT image; region one is the right diaphragmatic section (section  1 in the PCI-score), region two is the left diaphragmatic section (section  3 in the PCI score), region three is the liver hilum, region four is the area around the coeliac trunc, region five is the small bowel (section  9–12 in the PCI score), region six is pathological lymph nodes above the renal hilum, and region seven is enlarged CPLN. The short score gives a maximum of seven points (one point for each region with a positive finding). The short score evaluation was retrospectively analyzed by one radiologist with subspecialty training in gastroradiology (AK, 18 years of experience), and from the same CT image as CT-PCI and CPLN. A subset of 20 consecutive cases with images was independently re-read and scored (CT short score) by a second radiologist with subspeciality training in gastroradiology (HS, 13 years of experience). Three cases with CT images and short score ratings are shown in Fig. 1A/B/C/D.

### Tumor characteristics and clinical information

Tumors diagnosed 1991–2007 were re-evaluated by a senior pathologist (KJ) concerning histological subtype and histological grade [14]. From 2008 and onward, tumor information was extracted from the original pathology reports (reports now being more structured). No borderline tumors were included in the study, as none had been registered. Of the 166 OC cases (100%), 7 (4%) cases were defined as non-epithelial and excluded. Of the 159 OC cases eligible for analysis, the distribution was as follows: 99 (60%) were classified as serous, 27 (16%) endometrioid, ten (6%) mucinous, seven (4%) clear-cell, and 12 (7%) undifferentiated/adenocarcinoma NOS, and four cases (2%) had missing information on histologic subtype in medical charts/pathology reports. As in a previously published paper on this OC cohort [5], due to the small number of cases and for purposes of clinical relevance, histological type and grade were combined into one variable with the following classification: high-grade serous tumors and endometroid tumors (all grades) were grouped, and all other histological types combined in one group (including serous type with low or unknown grade). Tumor grade was divided into low or high, with the previous intermediate grade classified as high grade. Information on clinical stage was obtained retrospectively from the medical charts, following the standardized WHO classification of tumor staging and classified as stage I or advanced stage (II-IV). Baseline information (medication, height, weight, parity pattern, menopausal status) was gathered and registered at study inclusion by trained nurses.

### Statistics

Logistic regression was used to analyze CT short score in relation to histologic type/grade (binary) and clinical stage (binary), which yielded odds ratios (OR) and 95% confidence intervals (CI). Adjustments were made for age at diagnosis (continuous) and histological subtype/grade or clinical stage (when the variable was not an endpoint). Associations between CT short score and OC survival were analyzed using Cox proportional hazards analysis, yielding a HR with a 95%CI; adjustments were made for age at diagnosis, histological type/grade, and clinical stage. The proportional hazards assumption was confirmed using a log-minus-log plot. A *p*-value < 0.05 was considered statistically significant. For sake of comparison, logistic and Cox regression analyses with CT-PCI in relation to the above stated outcomes were re-calculated and included in Table 2 together with CT short score, although previously published [5].

**Table 2:** PCI and CT short score in relation to stage, grade, and OC specific survival

	Median (min–max)					
	Stage I	Advanced Stage	OR (95% CI)	*p*-val	ORAdj^a^(95% CI)	*p*-val
PCI	0 (0–8)	17 (0–39)	1.29 (1.10—1.53)	0.002	1.26 (1.07—1.49)	0.007
CTShort score	0 (0–2)	3 (0–7)	3.07 (1.61—5.82)	0.001	2.76 (1.42—5.38)	0.003
	Low grade	High grade	OR (95% CI)		ORAdj^b^(95% CI)	
PCI	2.5 (0–39)	14 (0–39)	1.02 (0.99—1.06)	0.170	1.02 (0.98—1.06)	0.434
CTShort score	1 (0–6)	2 (0–7)	1.13 (0.91—1.40)	0.280	1.03 (0.80—1.32)	0.813
	Alive	Dead from OC	HR (95% CI)		HRAdj^c^(95% CI)	
PCI	2.5 (0–39)	17.5 (0–39)	1.05 (1.02—1.07)	< 0.001	1.04 (1.01—1.07)	0.003
CTShort score	1 (0–7)	2.5 (0–6)	1.23 (1.08—1.39)	0.001	1.17 (1.01—1.35)	0.033

^a^Adjusted for age at diagnosis and histological type/grade

^b^Adjusted for age at diagnosis and stage

^c^Adjusted for age at diagnosis and stage and histological type/grade

Crude linear regression was used to analyze baseline risk factors in relation to CT-PCI (continuous) and CT short score (continuous). Baseline risk factors with a *p*-value < 0.2 in either CT-PCI or short score (i.e. parity (linear), nulliparity (binary), menopausal status (categorical)) were further analyzed in adjusted regression analyses (age at diagnosis, histological type/grade and clinical stage). In the subset analysis (*n* = 20 cases), relations between the CT short score from the two readers were illustrated descriptively (scatter plot) and calculated with Pearson correlation. Stata SE was used for the statistical analyses (version 16.0. College Station, Texas: StataCorp.).

## Result

**Fig. 2:**
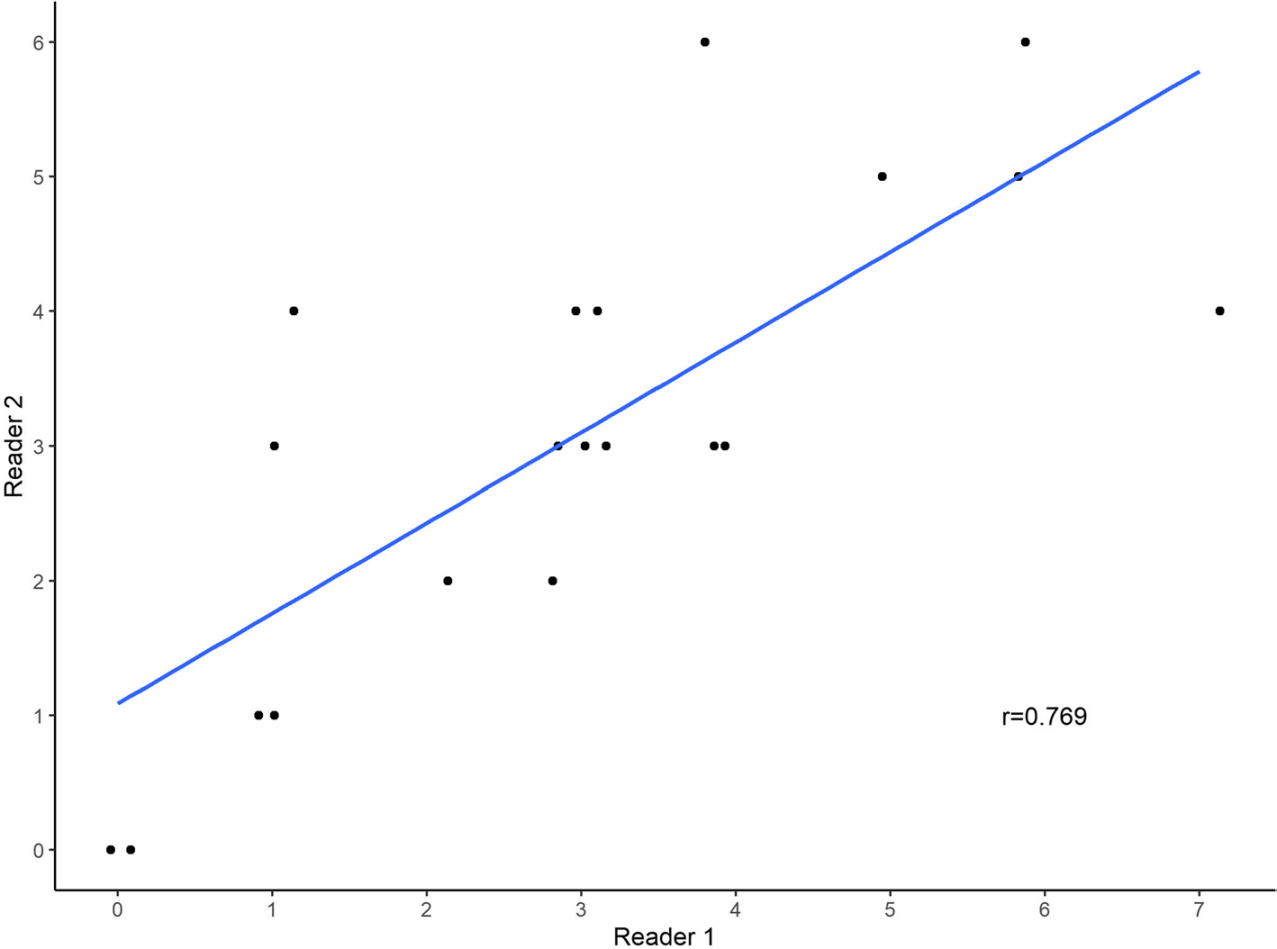
Scatterplot showing CT short scores from two independent readers, subset analysis (*n* = 20 cases)

The distribution of patient characteristics, tumor characteristics and CT parameters is illustrated in Table 1. As seen in the scatter plot and corresponding correlation coefficient (*r* = 0.769), there was a clear relation and high correlation between the CT short scores from the two readers (subset analysis *n* = 20 cases) (Fig. 2).

### CT short score in relation to OC prognostic factors

There was a statistically significant relation between increasing short score and advanced clinical stage (OR_adj_ 2.76 (1.42—5.38) *p* = 0.003), adjusted for age at diagnosis and histologic type/grade (Table 2). This relation was even stronger than the re-calculated and previously published (identical results) relation between CT-PCI and clinical stage. There was no significant association between short score and histologic type/grade (OR_adj_ 1.03 (0.80—1.32) *p* = 0.813). Lastly, higher short score was significantly associated with impaired OC specific survival (HRadj1.17 (1.01—1.35) *p* = 0.033), adjusted for age at diagnosis, histologic type/grade, and clinical stage. In comparison with CT-PCI, the short score demonstrates significant associations of similar or stronger magnitude.

### Base line OC risk factors in relation to short score and CT-PCI

In the initial explorative and crude regression analyses, three baseline factors presented with a *p*-value < 0.2 in either short score or CT-PCI (Table 3). These three baseline variables were further tested in adjusted analyses (age at diagnosis, subtype/grade, clinical stage) (Table 4).

**Table 3:** Baseline factors in relation to CT short score and PCI

	CT short score		PCI	
	β (95% CI)	*p*	β (95% CI)	*p*
Parity	0.34 (-0.01—0.70)	0.059	2.30 (0.11—4.49)	0.040
Nullipara = No	0.48 (-0.57—1.53)	0.366	4.29 (-2.17—10.76)	0.191
Oral contraceptives = Yes	-0.30 (-1.13—0.53)	0.481	-2.54 (-7.67—2.59)	0.328
Height, cm	0.04 (-0.03—0.11)	0.267	0.24 (-0.21—0.69)	0.284
Weight, kg	0.02 (-0.02—0.06)	0.291	0.10 (-0.15—0.36)	0.409
BMI	0.04 (-0.08—0.16)	0.511	0.15 (-0.59—0.88)	0.692
BMI (cat.)		0.394		0.684
< 25.00	Ref		Ref	
25.00–29.99	0.40 (-0.51—1.30)		2.40 (-3.24—8.04)	
≥ 30.0	0.93 (-0.58—2.44)		1.86 (-7.57—11.28)	
Menstrual status		0.226		0.123
Pre	Ref		Ref	
Peri	1.48 (-0.31—3.27)		7.75 (-3.26—18.76)	
Post	0.51 (-0.40—1.42)		5.40 (-0.20—11.00)	
HRT = Yes	-0.37 (-1.32—0.58)	0.438	-1.77 (-7.65—4.11)	0.552

**Table 4:**
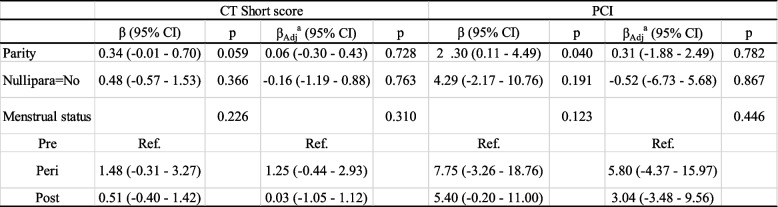
Parity, nulliparity status and menstrual status in relation to CT short score and PCI

^a^Adjusted for age at diagnosis and stage and histological type/grade

With increasing parity (i.e., increasing number of children born), there was no significant change in short score (β_adj_ = 0.06, *p* = 0.728) (Table 4). Results were similarly non-significant when analyzing parity in a binary fashion (nulliparity yes/no) (β_adj_ = -0.16, *p* = 0.763). Perimenopausal and postmenopausal women had a higher short score than premenopausal women, but the difference was not significant (overall *p* = 0.310 in adjusted analyses). Baseline characteristics in relation to CT-PCI did not show any significant associations for nulliparity status or menopausal status. When looking at the number of children, the CT-PCI increased for every child born (β = 2.30, *p* = 0.040), however after adjustment this effect could no longer be seen (β_adj_ = 0.31, *p* = 0.782).

## Discussion

In this study, we have identified strong relationships between a novel CT short score, advanced clinical stage, and impaired OC-specific survival. The short score, embracing both peritoneal carcinomatosis and CPLN, showed a similar or stronger relation to the OC outcomes than the previously studied CT-PCI in the same cohort [5]. From the radiologist’s perspective, this means that a pragmatic way of evaluating high risk CT parameters can be combined with a clinically trustworthy score.

The interpretation of the CT image is subjective, and the performance of CT in detecting tumor deposits varies between organs and regions [15]. With the attempt to structure CT-evaluation and reporting regarding peritoneal carcinomatosis, the potential implementation of CT-PCI in OC has been extensively studied. Previous work on CT-PCI, partly by our group on a more recent cohort, has shown good agreement between CT-PCI and surgical PCI [16, 17], a link between increased CT-PCI and increased risk of OC residual disease at surgery [6, 16] and impaired progression free survival [18], but a mixed association with overall survival [7, 18]. Rosendahl et al. concluded on surgical PCI, that selected regions rather than the full PCI was more predictive of a favorable prognosis [8]. From a radiological perspective, that would also be more feasible from a workflow perspective. This study shows that our novel CT short score on high-risk image parameters (CT-PCI and CPLN) performs even better than CT-PCI in terms of prognosis, and this has to the best of our knowledge not previously been studied. Another image factor of interest, although not available in this cohort, is CT-ascites, and previous work from our group has shown CT-ascites more than 1000 ml to be associated with high surgical PCI and residual disease [16]. Taken together, the fact that both CT-PCI and short score was strongly associated with advanced OC clinical stages is of high clinical relevance. It gives the possibility to identify patients, before surgery, with high tumor load and therefore high risk of incomplete cytoreductive surgery, that being known as the strongest prognostic factor in OC. Further studies with prospective analyses of CT-PCI, CT short score, CT-ascites, and clinical outcomes are however needed prior to a potential clinical implementation.

An aggressive cancer generates an aggressive CT image with high-risk image factors. Specific base line characteristics potentially leading up to such an image, or other adverse outcome [9], are worth highlighting in order to increase the understanding of the underlying biology illustrated in the image. Even if not modifiable, these factors may aid in creating radiological risk profiles, where images may be interpreted differently or with higher suspicion based on a woman’s risk profile. There are several established OC risk factors with family history being the most important [19], and with oral contraceptives having a protective role [20]. Regarding parity patterns, nulliparity [21] and infertility [22] have been shown to increase the risk of OC, but data on BMI and OC risk varies with menopausal status. However, there are no previous studies on OC risk factors and image findings. With a rather explorative approach, we detected three base line factors related to parity and menopausal status, possibly connected to CT-PCI and short score, but no association held true in adjusted analyses. There may be several reasons for this, but one limitation is the long time span (i.e., between study inclusion/risk factor collection and later OC diagnosis) wherein several factors may interact, and another reason may be the limited sample size. Larger studies are needed in order to identify differences in risk factor associations between the different image patterns (aggressive vs less aggressive), which in turn could improve our understanding of ovarian carcinogenesis.

Some methodological issues require consideration. The established CT-PCI score has been shown with high intra- and inter-observer reliability [3] and the CT short score can be seen as an abbreviated variant. In this present study, only one radiologist (and at one time point) interpreted the images, which is a shortcoming. However, a subset of 20 cases with abdominal CT images was re-read and scored (CT short score) by a second radiologist, showing high correlation between readers which we believe is a promising finding. However, to fully test the important clinical aspect of reliability between radiologists, two readers interpreting all cases independently is of the essence. At our institution, a prospective study analyzing CT short score and surgical short score is ongoing (ten women with ovarian cancer enrolled up until now), and a second radiologist reading all cases is planned for. We hope that such study will allow for further in-depth analyses regarding CT short score feasibility.

This is a retrospective study including cases over a long time span, and information on potential neoadjuvant therapy, surgical method, and surgical outcome was not part of the gathered clinical data. Adequate surgical cytoreduction is the most important independent factor affecting survival in epithelial OC and the lack of this parameter is especially noticeable. The survival analyses in this study were however adjusted for histological subtype/grade and clinical stage, both factors that strongly contribute to selecting the method of surgery and oncological treatment and thus may be seen as proxy variables for surgical method and outcome.

Our study has several clinical implications ranging from the general perspective to the very practical everyday work. An image-based short score would be an effective (for the radiologist) and comprehensive (for the gynecologist and oncologist) way of communicating important and aggressive image information in the setting of for instance a multidisciplinary conference. As of today, the structured surgical report from the European Society of Gynecological Oncology (ESGO) already includes surgical PCI, so the addition of CT-PCI or short score would be both logical and beneficial. As a future and visionary goal, the image short score could be even sharper if risk factor information could be used interpreting the image in dubious findings, and perhaps the presence or absence of a particular risk factor could help the radiologist deeming a particular finding as pathological or not. Taken together, imaging is central in OC care, and future research is needed.

In conclusion, we have identified strong relationships between an image short score and advanced clinical stages and impaired OC survival. A pragmatic approach (based on CT) to evaluate high risk image findings in OC could reduce the radiologist’s workload and at the same time provide structured reports to surgeons and oncologists involved in OC care.

## Data Availability

The datasets used and/or analysed during the current study are available from the corresponding author on reasonable request.

## Abbreviations

BMI: Body Mass Index
CPLN: Cardiophrenic Lymph Nodes
CT: Computer Tomography
CI: Confidence Interval
HR: Hazard Ratio
OC: Ovarian Cancer
PC: Peritoneal Carcinomatosis
PCI: Peritoneal Carcinomatosis Index
